# Impact of Housing and Community Conditions on Multidimensional Health among Middle- and Low-Income Groups in Hong Kong

**DOI:** 10.3390/ijerph15061132

**Published:** 2018-05-31

**Authors:** Jionghua Wang, Bo Huang, Ting Zhang, Hung Wong, Yifan Huang

**Affiliations:** 1 Institute of Space and Earth Information Science, The Chinese University of Hong Kong, Hong Kong, China; gubb6732@gmail.com (J.W.); tingzhang@link.cuhk.edu.hk (T.Z.); 2 Shenzhen Research Institute, The Chinese University of Hong Kong, Shenzhen 518057, China; 3 Department of Geography and Resource Management, The Chinese University of Hong Kong, Hong Kong, China; 4 Department of Social Work, The Chinese University of Hong Kong, Hong Kong, China; hwong@swk.cuhk.edu.hk; 5 Department of Psychology, University of British Columbia, Vancouver, BC V6T 1Z4, Canada; teafest@gmail.com

**Keywords:** housing conditions, community conditions, health outcomes, lasso, Hong Kong

## Abstract

With decades of urbanization, housing and community problems (e.g., poor ventilation and lack of open public spaces) have become important social determinants of health that require increasing attention worldwide. Knowledge regarding the link between health and these problems can provide crucial evidence for building healthy communities. However, this link has heretofore not been identified in Hong Kong, and few studies have compared the health impact of housing and community conditions across different income groups. To overcome this gap, we hypothesize that the health impact of housing and community problems may vary across income groups and across health dimensions. We tested these hypotheses using cross-sectional survey data from Hong Kong. Several health outcomes, e.g., chronic diseases and the SF-12 v. 2 mental component summary scores, were correlated with a few types of housing and community problems, while other outcomes, such as the DASS-21–Stress scores, were sensitive to a broader range of problems. The middle- and low-income group was more severely affected by poor built environments. These results can be used to identify significant problems in the local built environment, especially amongst the middle- and low-income group.

## 1. Introduction

Housing and community problems can significantly affect citizens’ health and well-being [1,2]. With decades of urbanization, these problems are increasingly occurring in urbanized areas that suffer from urban decay [3]. Local authorities have launched many projects, namely, urban renewal [4], to improve the living conditions of residents [5,6,7]. A profound understanding of the health impact of housing and community problems can aid in the identification of crucial problems in urban areas. Related projects and policies could eventually benefit local residents [8].

Empirical models are widely used to obtain an understanding of the relationships between social determinants (such as housing and community problems in this study) and individuals’ health outcomes. The results can be used to understand the mechanism by which a social determinant works. For example, Kraev examined the influence of second-hand smoke exposure on multiunit homes, and the empirical results indicated smoke contamination infiltration from a neighboring apartment [9]. Similarly, a recent study investigating the association among ventilation, air quality, and health revealed that weatherization can improve mental health in adults and alleviate several health problems (i.e., headaches, eczema, and skin allergies) in children [10]. Most previous studies investigating housing and community problems and their health impacts have been conducted based on a unidimensional health outcome framework. However, the impacts on multiple health outcomes are rarely discussed for these two social determinants. Most health impact assessment studies adopt a multidimensional (10.6 health impacts per approach) framework [11]. We recognize that knowledge about the impact on multidimensional health is necessary for addressing community and housing issues, and that unidimensional approaches can overlook some undetected but significant health determinants, and give an incomplete and misleading picture. Consequently, the health determinants of holistic health are under-considered. 

Nevertheless, compared with the health of the wealthy, the health of those in the middle- to low-income groups is more sensitive to environmental disadvantages [12,13]. This latter group is considered to have limited health equipment (e.g., tools for cleaning the house and clothing, personal hygiene products, and common medical supplies) and to be deficient in health-related behavior and knowledge [14]. The disparity in health status among income groups is considered an income-related health inequality [15,16]. Initially, this disparity was studied as a standalone phenomenon [17,18], but currently, it is considered a dynamic process in which health is substantially affected by social determinants [19]. Further, some studies point out that the association between health outcomes and these determinants among middle- and low-income groups is unique, i.e., there exists income-related heterogeneity [20,21]. However, studies investigating the heterogeneity among different income groups in terms of housing and community problems are limited.

In addition, Hong Kong, the area investigated in this research, is a typical highly urbanized region. Most people are economically forced to live in high-density communities and suboptimal apartments. The high housing and rent prices narrow the choices of housing and communities [22,23]. Consequently, affordably improving their housing and community conditions is challenging, especially for those in the middle- to low-income group, who usually have to live in public rental housing (PRH) [24] provided by the government after a strict application process and a long waiting list. Therefore, these citizens have limited opportunity to actively improve their living condition by purchasing or renting a newly designed apartment, because of their financial situation and the present public housing policy. They play a passive role in the improvement of their living condition. Notably, these citizens are vulnerable and more reliant on the government. Thus, an empirical study that can inform the local government on ways to improve living conditions is particularly crucial for the well-being of residents. However, few studies have explored the impact of poor community and housing conditions on health in this city. Evidence that can guide the local government in policymaking remains limited. Thus, establishing a reliable and general connection between the abovementioned social determinants (i.e., housing and community conditions) and health outcomes is necessary and urgent for this city. 

To fill the gap, this research aims to identify the connection between multidimensional health outcomes and the built environment (primarily housing and community conditions) among different income groups. We highlight the following three contributions of this study: (1) the association between social determinants (i.e., housing and community problems) and multidimensional health outcomes are conducted and cross-compared; (2) the role of income-related heterogeneity in this association is investigated; and (3) empirical knowledge regarding the health impact of the built environment in Hong Kong is obtained. Specifically, we investigated the impact of eight housing conditions and ten community problems on seven health outcomes (i.e., SF-12 Physical Health Composite Scores (PCS), SF-12 Mental Health Composite Scores (MCS), Depression Anxiety Stress Scales (DASS), self-rated general health status, chronic disease, and sleep problems). The empirical models were built based on a regression analysis with a cross-sectional survey dataset (*n* = 1785) and adjusted for age, occupation, accommodation type, educational attainment, gender, body mass index (BMI), and smoke and passive smoke exposure. It should be noted that this study intends to reveal the health impact disparity among different health measures and social groups, rather than a sophisticated connection between one specific built problem and its potential health outcomes. However, it will still elucidate how built environment problems affect citizens’ health and well-being in Hong Kong.

## 2. Data and Methods

### 2.1. Study Area

Hong Kong, a coastal city, is located in Eastern Asia. It has a population of 7.4 million people and a size of 2755 km^2^. Hong Kong is frequently considered a prosperous international financial center. However, it is also a crowded city (6544 people/km^2^) with a limited living environment [25]. Severe income inequality and high housing prices prevent many people from obtaining decent housing [26]. Accordingly, the local government considers the citizens’ housing problem amongst the most critical livelihood issues, and is dedicated to improving this problem via a series of policy interventions, e.g., PRH [24], and the home ownership scheme (HOS) [27]. However, the marginal cost of improving housing or community conditions by moving is usually high, and can be overwhelming, particularly for low- to middle-income households [28,29,30]. In addition, dwellers play a passive role in improving their built environment. People who are forced to tolerate community or housing problems experience lower well-being. Thus, the local government is obligated to identify the negative impacts and subsequently improve these conditions. 

### 2.2. Survey Data

We used the data from the first wave of the Strategic Public Policy Research (SPPR) project called “Trends and Implications of Poverty and Social Disadvantages in Hong Kong: A Multi-disciplinary and Longitudinal Study” [31]. The data were collected between May 2014 and July 2015 by Policy 21 Limited. The data were collected in face-to-face interviews, with a response rate of 60.2%. The sample included 1980 households with at least one adult (aged 18 or older). One adult was randomly selected to represent his or her household. We excluded some participants with missing values in one or more fields. Finally, 1785 observations were used in the analysis.

#### 2.2.1. Sample Characteristics

Most respondents were female (58.26%), and this ratio was even higher in the low- to middle-income group (60.55%). Approximately half (49.19%) of the respondents were born in Hong Kong, and held a lower-secondary degree or below (53.28%). The equalized household income was 13,405.33 HKD, and the equalized household income of the low- to middle-income group was 8888.55 HKD (33.69% lower) (Equalized household income is household income equalized by household size. In this study, it is calculated as $E=I/\sqrt{P}$, where E is the equalized household income (HKD), I is the total household income (HKD), and P is household size.). More than half of the respondents lived in PRH (56.63% and 64.90% of the low- and middle-income groups), and the respondents’ ages followed a distribution similar to that of the whole population, with a mean value of 51.60 years. The two most prevalent occupations were “service and sales workers” (11.82%) and “elementary occupations” (19.27%). 

#### 2.2.2. Health Measures

Health is inherently a multidimensional concept. However, health indicators are usually designed to reflect a person’s health from one specific perspective. The components of a person’s holistic health status, such as physical health, mental health, and social health, are likely to be measured separately, and with a target perspective. Health outcomes in relation to urban management or policymaking should better reflect general health and well-being; i.e., the outcome should be more representative of the population’s holistic health. Thus, this study includes seven indicators (Table 1) covering different dimensions, thus offering a more comprehensive understanding of how holistic health is affected by community and housing problems. Furthermore, the differences and commonalities among these indicators can be cross-compared, which may outperform some approaches based purely on unidimensional health measures. We include the following health outcomes, and Table 1a,b describes their characteristics.

- SF-12 v. 2 [32,33] (PCS; MCS) is a short-form instrument consisting of 12 questions. It is a multipurpose health scale that is comprehensive, readily available, and psychometrically sound for a large-population survey. This scale covers dimensions such as physical functioning, physical roles, bodily pain, general health, vitality, social functioning, emotional roles, and mental health. The PCS and MCS range from 0 to 100, and people who have higher scores are considered healthier.
- Self-rated health [34] is widely used in evaluations of a person’s overall health status [35]. In this study, people who considered their health “excellent”, “very good” or “good”, as opposed to “fair” or “poor”, are considered healthy. While this measure is argued to be subjective [36], it remains valuable to reflect holistic health and to supplement some objective measures.
- Chronic disease can be affected by housing conditions and community environments [36,37]. As the disease of the 21st century, it not only impairs the health of residents, but also places a heavy burden on local financial and healthcare systems. Chronic disease is especially relevant in Hong Kong considering that it is an aging society. Thus, because aging people are at high risk, exploration of chronic disease should be prioritized. In this study, we included four measures of chronic disease (i.e., having one or more kinds of chronic disease, hypertension, high blood cholesterol and diabetes).
- Sleep problems can exacerbate many clinical conditions [38]. Moreover, people who live in highly urbanized regions, such as Hong Kong, are exposed to more stress and a noisy environment. However, sleep problems are not explicitly represented in most health indicators. While sleep problems might result from sets of housing problems and community issues, studies have rarely explored the relationship between sleep problems and the aforementioned issues. Thus, we include sleep problems in this approach. People who claim to have “very good” or “fairly good” sleep are considered to not have a sleep disorder, whereas those who report having “fairly bad” or “very bad” sleep are.
- DASS-21 (the Depression Anxiety Stress Scales) is composed of 21 self-report items and is designed to identify emotional disturbances (anxiety and stress in this case) [39,40]. DASS-21 was developed by researchers at the University of New South Wales. This measure is widely used as a health indicator for mental health, and people who have higher scores tend to have more severe corresponding symptoms.

#### 2.2.3. Community/Housing Problems and Control Variables

In this study, we included eight housing conditions and ten community problems, as shown in Table 2. These items were proposed by residents in advance, and were subsequently measured in face-to-face interviews. Respondents who reported one problem were assigned a 1 in the corresponding category, and those who did not report a problem were assigned a 0. Almost all residents reported one or more housing and/or community problems in the survey. However, the incidence of most problems was low (approximately 5~10%), and only 5 out of 18 problems were reported in more than 10% of households.

To remove confounding effects, we controlled for the informants’ age, gender, household income, occupation, educational attainment, BMI, smoke exposure and accommodation type. Formally, most variables were converted into dummy variables with one reference group, as shown in Table 2.

### 2.3. Statistical Analysis

The statistical analysis was performed to identify the relationships between the health measures and the built environment. We employed a lasso regression for the variable selection and performed a set of OLS/logistic regressions based on the lasso results. Since most explanatory variables were dummy variables or in a binary form, we performed a generalized lasso linear regression [41,42] to address the multicollinearity [43]. The least absolute shrinkage and selection operator, namely, lasso, was first introduced by Robert Tibshirani in 1996 [44] and is well-known for addressing covariate variables, such as the dummies in this study, while providing greater accuracy and more interpretable results. For each health measure, the model with penalty coefficient λ, which can yield the lowest error, was selected as the optimal model, and corresponding variables with remaining (β≠0) coefficients were included in the following modeling. We performed linear regression taking PCS, MCS, DASS—Anxiety and DASS—Stress as response variables and logistic regression for self-reported health, chronic disease, and sleep problems, with selected variables from the former generalized lasso regressions. The abovementioned analyses were performed using MATLAB 7.0.

### 2.4. Analytical Methods

This research attempted to investigate the influence of community and housing conditions on multiple health outcomes among different income groups. First, to test the hypothesis that there are differences between the whole population and the middle- to low-income group, we generated the following two subdatasets: Data-A included all participants (*n* = 1785), and Data-B included only the middle- to low-income participants (*n* = 1262, those with an equalized household income lower than 15,500 HKD/month, which is the median simultaneous personal income). Next, for each dataset (*n* = 2), we conducted several regression models involving a set of health measures (*n* = 7). In particular, we used 14 (2 datasets * 7 health measure) regression models, in which one health measure was used as the response variable and community/housing problems were used as the explanatory variables along with a set of control variables (age group, age, occupation, accommodation type, educational attainment, gender, BMI, and smoke and passive smoke exposure). For each model, a lasso regression was conducted for the variable selection. Then, according to the lasso regression, the selected variables were included in the OLS/logistic regressions. Finally, we conducted seven regression models of each income group, for a total of 14 models. Given the regression coefficients, we compared the impact of housing and community problems on different health outcomes. Furthermore, the difference between the middle- to low-income group and the whole population was explored by comparing the type and strength of the significant statistical problems between the two subdatasets.

## 3. Results

Generally, health outcomes were affected by different social determinants. Even one determinant that had a significant impact on two or more health outcomes had strengths that differed based on the standardized coefficients. Table 3a,b shows the regression coefficients of the whole population and the middle- and low-income group. For each column, it shows the result of one regression model that consists of a response variable (e.g., PCS and MCS) on the top, followed below by the coefficients of housing and community conditions as explanatory variables. For each row, it lists one housing and community condition with its name and the coefficients in different regression models. In one column, an omitted coefficient marked as blank in Table 3 indicates that its corresponding housing and community issue, as an independent variable, does not pass the variable selection check in the former lasso regression. The selected variables are then displayed with their coefficients and confidence interval (95%). The variables (housing and community in the table) associated with one health measure are labeled with *, **, or ***, indicating different statistically significant levels.

### 3.1. Results of the Whole Population

The PCS were related to conditions such as shortage of space (*p* < 0.01), rot in window frames or floors (*p* < 0.001), light pollution (*p* < 0.05), poor street lighting or broken pavement (*p* < 0.01), and risk from traffic to pedestrians and cyclists (*p* < 0.05). The MCS were lower by approximately 2.4 points among local residents who reported a problem with drunk or rowdy people in the street/park (*p* < 0.01) and by 1.8 points among residents who reported a problem with poor ventilation (*p* < 0.05). The DASS—Anxiety scores were higher among residents living in housing with rot in the window frames or floors (*p* < 0.001) and poor ventilation (*p* < 0.05), and in communities with traffic or business noise (*p* < 0.01), noisy neighbors or loud parties (*p* < 0.05), criminal activity (*p* < 0.01), and drunk or rowdy people in the street/park (*p* < 0.01). The DASS—Stress scores were associated with the following conditions: rot in window frames or floors (*p* < 0.05), poor ventilation (*p* < 0.001), light pollution (*p* < 0.05), traffic or business noise (*p* < 0.05), noisy neighbors or loud parties (*p* < 0.0001), illegal parking (*p* < 0.05), drunk or rowdy people in the street/park (*p* < 0.01), criminal activity (*p* < 0.01), and problems with communal areas (*p* < 0.0001). Self-reported health status was related to shortage of space (*p* < 0.05), air pollution (*p* < 0.05), and noisy neighbors or loud parties (*p* < 0.05). The aforementioned problems were not significantly associated with chronic disease in this study. Sleep problems more frequently occurred with shortage of space (*p* < 0.01), light pollution (*p* < 0.05), and criminal activity (*p* < 0.05).

In summary, the different correlations among the health outcomes and community and housing problems potentially indicate that a unidimensional approach can be problematic, since the revealed correlated social determinants are incomplete. By contrast, multiple health outcomes can provide a more comprehensive picture of health outcomes. A similar phenomenon was observed in the middle- to low-income group. In the following section, we highlight some differences.

### 3.2. Results of the Middle- to Low-Income Group

The middle- to low-income group is considered more vulnerable when facing these problems. Hence, individuals in this group potentially follow a different mechanism than the whole population. To confirm this hypothesis, this study generated a subdataset representing this group and investigated the difference between this group model and the whole population model. We observed the following major discrepancies:

- The middle- to low-income group was sensitive to being too hot in the summer/too cold in the winter (*p* < 0.05) and to noisy neighbors or loud parties (*p* < 0.05) in terms of chronic diseases, while the whole population showed no sensitivity to any conditions related to this indicator.
- The middle- to low-income group was sensitive to fewer community/housing conditions (22 of 126 items) than their peers (28 of 126 items).
- The middle- to low-income group was more severely affected when reporting one poor condition or problem than the whole population; i.e., the absolute values of the significant items’ coefficients were larger. Thus, the health outcome in this group was affected to a greater extent under poor conditions.

## 4. Discussion

Our results demonstrate that the impact factors of health vary among measured dimensions. For instance, chronic disease was not related to any problem, while the DASS—Stress was correlated with nine housing/community problems. A unidimensional health measure may omit some critical social determinants of residents’ holistic health outcomes and provide limited information. Potential policy implementations for achieving environmental improvement could be partially guided by these results, and this study can prevent the implementation of a public health policy that demands a systematic exploration of the consequences of policy intervention [45].

We simultaneously modeled the influence of community and housing conditions on the whole population and the middle- to low-income group. Both the sensitive items and their sensitivity levels varied among these two subdatasets. Specifically, the middle- to low-income group’s health indicators were affected by fewer community and housing problems. However, each significant item had a more powerful influence on health status, since the coefficients’ absolute values were higher in the middle- to low-income group model than in the whole population model. The middle- to low-income group proportionally reported more housing conditions (6 out of 8: shortage of space, too hot in the summer/too cold in the winter, rot in window frames or floors, poor ventilation, rats or insects, and light pollution) and fewer community problems (6 out of 10: poor street lighting or broken pavement, risk from traffic to pedestrians and cyclists, illegal parking, drunk or rowdy people in the street/park, criminal activity, and problems with communal areas). A difference was observed between these two groups. Thus, applying or assuming a homogenous model including both groups is problematic, particularly for policymakers aiming to improve the living conditions of a specific vulnerable group (e.g., middle- to low-income in this study) [45]. These results identify the crucial problems of the middle- to low-income group with a higher precision. This study can inform the government about preventing income inequality from becoming a health inequality [46], particularly if the government aims to favor the middle- to low-income group in policymaking. In this case, our results regarding the identified health-related housing and community problems can help the Hong Kong government refine their measures to improve the living environment (Owners’ Corporation Formation Subsidy [47]) with a detailed aid list that may include only those significant housing and community problems. It will make the existing policy more economically efficient and more aligned with public interests. At present, the government measure does not consider the costs and benefits in terms of health outcomes. Moreover, it supports the improvement of all kinds of housing with equal strength, even though some conditions are less important or even irrelevant to citizens’ health, according to this study. Thus, by considering only those significant housing and community conditions, we may provide a shorter but targeted list of aid for the government.

The dimensions measured in previous studies were usually proposed based on the researchers’ prior knowledge, and several potential housing and community problems were omitted from the experimental design. These studies may fail to identify locally relevant problems in the built environment. In contrast, we investigated health outcomes based on community and housing problems reported by residents. This method constructed a more targeted and general list of housing and community conditions. Our results constitute a better generic guide for improving the local built environment. However, the use of self-reported community and housing conditions presents two limitations. First, only poor conditions were included as influencing factors, and the positive impacts of several pleasant landscapes were omitted. The impact of housing and community conditions can be better understood by including these positive impacts. Second, certain conventional measures of the built environment (e.g., accessibility to green space and walkability) were not included in this study. Thus, comparing our results with studies that have adopted these measures to account for the built environment is challenging. Further investigation is required to determine how multiple health outcomes are affected by a built environment more comprehensively.

We address the limitations of this study with the following three points. First, the analysis was based on typical cross-sectional data. The exposure and outcomes were simultaneously assessed. Consequently, the influence of condition improvement and the temporal relationship were not considered. To overcome the abovementioned limitation, we could employ a longitudinal dataset to clarify the causality between housing and community conditions and health outcomes in a future study. Second, the measure may suffer from certain error due to self-reporting bias given that the built environment was measured using a self-reported questionnaire. In a future study, the self-report items (health status and housing and community conditions) can be validated by accounting for contemporary objective measures, e.g., the service level of local facilities and the overall health status of the population. In practice, we recommend combining and cross-comparing multisource data (e.g., survey data, government statistical reports, social media data, and remote sensing data). Finally, we compared the difference between the middle- to low- income group and the whole population, and found that the whole-population approach might poorly identify certain housing and community problems. Moreover, these two datasets overlapped. Therefore, a comparison between their regression results is potentially statistically problematic.

## 5. Conclusions

In the present study, we modeled the relationship between a poor built environment and multidimensional health among different income groups in Hong Kong. This study complements prior studies that have demonstrated that housing and community conditions are relevant to the health of local residents [2,35,37]. This study also extends upon studies that focus predominantly on unidimensional health to reflect more holistic health. We built exclusive models of the residents and vulnerable groups, and examined the impact of housing and community conditions on multiple health outcomes. The present study can be described as an attempt to reveal the aforementioned heterogonous influence on different social groups and health measures. The results provide substantial impetus for the Hong Kong government to improve the built environment. For other cities, the results of this study may be useful in two ways. First, it verifies the importance of considering the differences among social groups and health measures in policymaking for cities that also have such problems as urban decay. Second, it may support those rapidly urbanizing cities in their planning by identifying potential critical housing and community problems that may arise in the future. From the perspective of the research method, this study suggests incorporating a cross-comparison among health measures with conventional unidimensional health measures. Our findings also reinforce the importance of considering the differences among social groups and their potential influence on policymaking.

A potentially significant problem is the differences among social groups, even across regions. This study rudimentarily demonstrated that the middle- to low-income group is particularly influenced by poor built environments. However, we still have insufficient knowledge regarding this problem in terms of whether, how, and the degree to which health outcomes are affected when a community or housing problem occurs in our study area. Further research can provide a more precise answer to this question using locally estimated or innovative survey data.

## Figures and Tables

**Table 1:** (a) Health measures among the whole population. (b) Health measures among the middle- to low-income group.

(a)				
Population	Max	Min	Average	SD
PCS	70.36	6.85	51.08	8.51
MCS	80.68	10.76	54.81	8.46
DASS—Anxiety	19.00	0.00	1.00	2.34
DASS—Stress	20.00	0.00	1.30	2.91
	Yes (* = 1)	No (* = 0)	**Positive Rate**	
Self-reported Health Status	1147	638	64.26%	
Chronic Disease	494	1291	27.68%	
Sleep Problems	513	1272	28.74%	
**(b)**				
**Middle/Low-Income Group**	**Max**	**Min**	**Average**	**SD**
PCS	69.02	6.85	50.56	8.92
MCS	72.61	17.98	53.88	8.55
DASS—Anxiety	19.00	0.00	1.10	2.48
DASS—Stress	20.00	0.00	1.39	3.04
	Yes (* = 1)	No (* = 0)	**Positive Rate**	
Self-reported Health Status	769	493	39.06%	
Chronic Disease	382	880	69.73%	
Sleep Problem	396	866	68.62%	

**Table 2:** Controlled variables and community/housing problems.

Variable		Population	Middle-/Low-Income	Gap *
Age Group	18–24	8.63%	7.27%	−15.73%
	25–34	10.31%	6.96%	−32.53%
	35–54 (ref)	29.41%	39.29%	33.59%
	55–64	21.40%	16.92%	−20.95%
	65–74	18.26%	14.39%	−21.21%
	75+	11.99%	14.94%	24.61%
Occupation	Managers and administrators	2.97%	1.66%	−44.11%
	Professionals	2.18%	0.79%	−63.74%
	Associate professionals	5.77%	2.77%	−52.05%
	Clerical support workers	9.19%	7.67%	−16.56%
	Service and sales workers	11.82%	11.70%	−1.02%
	Craft and related workers	9.47%	9.72%	2.67%
	Plant and machine operators and assemblers	3.59%	3.56%	−0.91%
	Elementary occupations	19.27%	21.74%	12.81%
	Other	0.22%	0.24%	7.80%
Living in Public Housing		56.53%	64.90%	14.81%
Educational Attainment	Primary and below	30.20%	35.42%	17.27%
	Lower secondary	23.08%	26.80%	16.11%
	Upper secondary	31.20%	28.22%	−9.55%
	Higher education (ref)	14.96%	8.77%	−41.35%
Born in Hong Kong		49.19%	41.19%	−16.27%
Gender	Male	41.74%	39.45%	−5.49%
	Female (ref)	58.26%	60.55%	3.93%
Housing Conditions	Shortage of space	21.57%	22.77%	5.55%
	Too hot in the summer/too cold in the winter	18.99%	20.55%	8.23%
	Damp walls, ceilings, floors, etc.	28.96%	28.14%	−2.82%
	Rot in window frames or floors	6.33%	6.88%	8.65%
	Problems with plumbing, drains or water supply	6.83%	6.48%	−5.09%
	Poor ventilation	8.35%	8.85%	6.03%
	Rats or insects	18.49%	19.76%	6.88%
	Light pollution	0.95%	1.11%	16.50%
Regional Problems	Poor street lighting or broken pavement	5.38%	4.51%	−16.25%
	Noise (traffic or businesses)	15.69%	16.60%	5.80%
	Noisy neighbors or loud parties	8.52%	9.01%	5.77%
	Air pollution	9.13%	9.49%	3.90%
	Lack of open public spaces	4.37%	4.51%	3.11%
	Risk from traffic to pedestrians and cyclists	2.52%	1.98%	−21.58%
	Illegal parking	5.38%	4.51%	−16.25%
	Drunk or rowdy people in the street/park	4.59%	4.58%	−0.11%
	Criminal activity	4.71%	4.58%	−2.65%
	Problems with communal areas	5.15%	5.14%	−0.23%
BMI	Underweight (<18)	10.14%	10.83%	6.81%
	Normal weight (18–25 ref)	59.72%	58.02%	−2.84%
	Overweight (25–30)	25.21%	26.09%	3.48%
	Obese (>30)	4.93%	4.82%	−2.19%
Smoke exposure	Never smoker (ref)	81.12%	80.08%	−1.28%
	Ex-smoker	4.48%	5.14%	14.70%
	Smoker	14.23%	14.39%	1.11%
Second-hand smoke in the household	None of time (ref)	75.35%	75.97%	0.82%
	A little/some of the time	16.36%	16.68%	1.96%
	All/most of the time	3.42%	3.32%	−2.92%
Second-hand smoke in office	None of the time (ref)	83.08%	84.82%	2.10%
	A little/some of the time	8.96%	8.22%	−8.24%
	All/most of the time	3.31%	3.16%	−4.47%
Equalized household income (HKD, mean)		13,405.33	8888.55	−33.69%

* $\mathrm{Gap}\;=\;\frac{P\left(middle-\;and\;low-income\right)-P\left(Population\right)\;}{P\left(Population\right)}$.

**Table 3:** (a) Impact of housing and community problems on the whole population (results of a linear/logistic regression). (b) Impact of housing and community problems on the middle- to low-income group (results of a linear/logistic regression).

(a)								
Housing and Community Conditions		Health Indicators—β(95% CI) *sig*.						
		PCS	MCS	DASS—Anxiety	DASS—Stress	Self-Reported Health	Chronic Disease	Sleep Problems
Housing Conditions	Shortage of space	−1.32 (−2.29, 0.35) **		0.08 (−0.19, 0.35)		−0.37 (−0.63, 0.12) **		0.38 (0.11, 0.65) **
	Too hot in the summer/too cold in the winter		−0.26 (−1.35, 0.83)	0.14 (−0.15, 0.43)		−0.05 (−0.35, 0.25)	0.3 (−0.02, 0.62)	
	Damp walls, ceilings, floors, etc.	−0.54 (−1.5, 0.42)	−0.29 (−1.23, 0.65)		0.16 (−0.17, 0.49)	−0.21 (−0.46, 0.05)	0.08 (−0.21, 0.36)	
	Rot in window frames or floors	−2.85 (−4.54, 1.17) ***		0.81 (0.34, 1.29) ***	0.75 (0.15, 1.35) *	0.32 (−0.13, 0.78)		
	Problems with plumbing, drains or water supply		−1.22 (−2.8, 0.35)	0.36 (−0.09, 0.82)	0.47 (−0.09, 1.03)		0.09 (−0.37, 0.55)	0.23 (−0.2, 0.65)
	Poor ventilation		−1.85 (−3.32, 0.39) *	0.49 (0.09, 0.9) *	0.96 (0.47, 1.46) ***	−0.14 (−0.52, 0.25)		0.09 (−0.31, 0.49)
	Rats or insects	1.09 (−0.06, 2.24)			−0.28 (−0.67, 0.11)	−0.24 (−0.54, 0.05)	0.07 (−0.27, 0.41)	0.16 (−0.14, 0.47)
Community Problems	Light pollution	−4.38 (−8.37, 0.39) *	−1.88 (−5.86, 2.1)	1 (−0.1, 2.11)	1.66 (0.31, 3.02) *	−0.84 (−1.94, 0.27)	0.61 (−0.47, 1.69)	1.55 (0.42, 2.69) **
	Poor street lighting or broken pavement	−2.53 (−4.3, 0.75) **					0.18 (−0.33, 0.69)	
	Noise (traffic or businesses)	−0.8 (−1.94, 0.35)		0.48 (0.18, 0.79) **	0.5 (0.1, 0.89) *		0.09 (−0.24, 0.43)	0.16 (−0.16, 0.47)
	Noisy neighbors or loud parties		−1.37 (−2.8, 0.06)	0.49 (0.09, 0.89) *	1.32 (0.83, 1.82) ***	−0.36 (−0.73, 0.01) *	0.39 (−0.02, 0.79)	
	Air pollution	−0.6 (−2.06, 0.85)	−0.36 (−1.75, 1.03)		−0.34 (−0.84, 0.16)	−0.36 (−0.72, 0.01) *	0.16 (−0.25, 0.57)	0.1 (−0.31, 0.5)
	Lack of open public spaces	−1.03 (−3.05, 0.99)			0.6 (−0.09, 1.28)			
	Risk from traffic to pedestrians and cyclists	−3.14 (−5.75, 0.53) *			0.39 (−0.51, 1.28)		−0.35 (−1.19, 0.48)	
	Illegal parking		−1.28 (−3.03, 0.48)		−0.63 (−1.24, 0.01) *	−0.25 (−0.72, 0.23)	−0.45 (−1.01, 0.11)	
	Drunk or rowdy people in the street/park	−1.3 (−3.24, 0.65)	−2.43 (−4.37, 0.49) *	0.82 (0.28, 1.36) **	0.94 (0.28, 1.61) **	0.32 (−0.21, 0.86)		
	Criminal activity		−1.43 (−3.28, 0.43)	0.79 (0.28, 1.3) **	0.94 (0.3, 1.57) **	−0.44 (−0.92, 0.05)	0.36 (−0.15, 0.87)	0.59 (0.11, 1.08) *
	Problems with communal areas	−0.69 (−2.49, 1.1)	−0.89 (−2.69, 0.9)	0.25 (−0.25, 0.75)	1.47 (0.85, 2.08) ***	−0.37 (−0.84, 0.09)	0.27 (−0.24, 0.77)	0.19 (−0.29, 0.67)
**(b)**								
**Housing and Community Conditions**		**Health Indicators—β(95% CI)** ***sig*.**						
		**PCS**	**MCS**	**DASS—Anxiety**	**DASS—Stress**	**Self-Reported Health**	**Chronic Disease**	**Sleep Problems**
Housing Conditions	Shortage of space	−1.45 (−2.61, 0.29) *		0.22 (−0.12, 0.55)		−0.34 (−0.62, 0.05) *		0.41 (0.1, 0.73) **
	Too hot in the summer/too cold in the winter			0.21 (−0.16, 0.57)			0.38 (0.02, 0.75) *	
	Damp walls, ceilings, floors, etc.		−0.48 (−1.65, 0.69)			−0.17 (−0.46, 0.12)	0.1 (−0.23, 0.43)	
	Rot in window frames or floors	−3.13 (−5.18, 1.08) **		0.96 (0.41, 1.52) ***	0.81 (0.12, 1.51) *			
	Problems with plumbing, drains or water supply	1.79 (−0.3, 3.88)	−1.4 (−3.32, 0.52)		0.52 (−0.19, 1.22)			0.23 (−0.3, 0.75)
	Poor ventilation		−1.39 (−3.1, 0.31)	0.34 (−0.17, 0.85)	0.77 (0.18, 1.35) **	−0.27 (−0.71, 0.17)		
	Rats or insects		−1.13 (−2.5, 0.24)			−0.39 (−0.72, 0.07) *	0.15 (−0.25, 0.55)	0.26 (−0.11, 0.62)
Community Problems	Light pollution	−4.36 (−8.88, 0.17)			0.82 (−0.72, 2.36)		0.62 (−0.54, 1.78)	1.42 (0.16, 2.68) *
	Poor street lighting or broken pavement	−2.02 (−4.4, 0.35)						
	Noise (traffic or businesses)	−0.25 (−1.64, 1.13)		0.53 (0.13, 0.93) **	0.51 (0.06, 0.97) *			0.26 (−0.09, 0.62)
	Noisy neighbors or loud parties		−1.57 (−3.24, 0.1)	0.58 (0.09, 1.07) *	1.44 (0.85, 2.03) ***	−0.46 (−0.87, 0.04) *	0.52 (0.07, 0.97) *	0.24 (−0.22, 0.69)
	Air pollution	−1.3 (−3.07, 0.47)	−0.64 (−2.31, 1.02)	0.06 (−0.45, 0.57)		−0.38 (−0.8, 0.03)	0.21 (−0.24, 0.67)	
	Lack of open public spaces				0.31 (−0.49, 1.11)		−0.43 (−1.13, 0.28)	
	Risk from traffic to pedestrians and cyclists	−1.87 (−5.51, 1.76)						−0.61 (−1.69, 0.46)
	Illegal parking	−0.5 (−2.95, 1.94)					−0.65 (−1.37, 0.07)	−0.52 (−1.24, 0.2)
	Drunk or rowdy people in the street/park	−1.42 (−3.83, 1)	−3.53 (−5.83, 1.22) **	1.19 (0.51, 1.87) ***	1.29 (0.47, 2.11) **			
	Criminal activity		−0.69 (−2.98, 1.61)	0.53 (−0.14, 1.19)	0.8 (0.01, 1.58) *			0.74 (0.14, 1.33) *
	Problems with communal areas	−1.15 (−3.4, 1.1)			1.49 (0.73, 2.25) ***		0.27 (−0.31, 0.86)	0.39 (−0.18, 0.96)

Notes: * *p* < 0.05, ** *p* < 0.01, *** *p* < 0.001. Conditions that were not significant in the former lasso regression are excluded and left blank. All models were adjusted for age, occupation, accommodation type, educational attainment, gender, BMI, and smoke and passive smoke exposure.
